# Stress-Specific Carbonylation and Proteasome 20S Activity in Potato Under Drought, Elevated Temperature, and Combined Stresses: Linking Oxidative Damage to Proteome Regulation

**DOI:** 10.3390/plants15060939

**Published:** 2026-03-19

**Authors:** Dominika Boguszewska-Mańkowska, Justyna Fidler-Jarkowska, Marta Gietler, Małgorzata Nykiel

**Affiliations:** 1 Plant Breeding and Acclimatization Institute—National Research Institute in Radzików, Jadwisin Division, Department of Potato Agronomy, Szaniawskiego Str. 15, 05-140 Serock, Poland; 2 Department of Biochemistry and Microbiology, Institute of Biology, Warsaw University of Life Sciences-SGGW, Nowoursynowska 159, 02-776 Warsaw, Poland

**Keywords:** *Solanum tuberosum* L., drought stress, elevated temperature, protein carbonylation, 20S proteasome, agronomic parameters

## Abstract

Drought and elevated temperature are major abiotic stresses that limit potato growth and productivity; however, their combined effects on biomass and oxidative damage to proteins remain poorly understood. We investigated individual and interactive effects of drought and elevated temperature on growth traits, yield, protein carbonylation, 20S proteasome activity, and the leaf proteome. Results show that while an elevated temperature alone did not significantly impair vegetative biomass or yield, it markedly intensified the negative impacts of drought during simultaneous exposure. Drought and combined stress substantially reduced stem and leaf mass, as well as assimilation area. Biochemically, drought induced protein carbonylation and stimulated 20S proteasome activity. Interestingly, elevated temperature reduced carbonylation and proteasome activity, yet its presence in combined stress exacerbated oxidative damage compared to drought. Proteomic analysis revealed stress-specific carbonylation of molecular chaperones, antioxidant enzymes, and proteins involved in photosynthesis, glycolysis, and energy metabolism. These results suggest that while potato plants exhibit resilience to moderately elevated temperature, the synergistic effect of heat and drought triggers a more severe oxidative challenge. This requires enhanced proteolytic and antioxidant mechanisms to maintain growth and productivity under complex stress conditions.

## 1. Introduction

The unique developmental characteristics of potato (*Solanum tuberosum* L.) plants play a key role in their resilience and adaptive capacity [1]. Potatoes simultaneously produce photosynthetically active organs (leaves and stems) and underground storage organs (tubers), giving rise to complex physiological interactions between above- and below-ground tissues. This functional duality underlies dynamic source–sink relationships that determine the allocation of assimilates and biomass distribution during plant development [2].

When subjected to environmental stresses such as drought, flooding, or elevated temperature, potatoes activate stress perception and metabolic regulatory mechanisms that enable resource prioritization. For example, water deficit may limit shoot growth while promoting energy allocation toward tuber development [3]. Such stress-induced shifts reflect profound alterations in source–sink relationships, assimilate partitioning, and carbon and energy metabolism, ultimately influencing plant growth and yield [4]. These processes are tightly regulated at the molecular level, and disturbances often manifest as changes in protein abundance, stability, and post-translational modifications [5].

These molecular responses can be directly linked to whole-plant performance, which is assessed using agronomic and physiological parameters such as biomass accumulation, leaf and stem mass, leaf area, and yield. By linking these phenotypic measurements with proteomic data, it is possible to connect molecular stress responses with the plant’s ability to maintain water balance and resource allocation [6,7].

The potato is globally significant due to its agricultural importance and its role as a major crop model for studying plant responses to abiotic stress. This crop is highly sensitive to water deficits and elevated temperature, making it an ideal model system for investigating stress-induced changes across multiple levels of plant organisation. The considerable genetic and phenotypic diversity among potato cultivars offers opportunities to identify traits and molecular mechanisms associated with stress tolerance [8].

At the molecular level, oxidative stress plays a critical role in the plant’s reaction to drought and elevated temperature [9]. Under these conditions, plants may produce excess reactive oxygen species (ROS), leading to oxidative damage to lipids, nucleic acids, and proteins. Proteins are particularly vulnerable to oxidative modification, with carbonylation being one of the most prevalent and irreversible forms of damage. This modification can impair protein function, promote degradation, and disrupt metabolic pathways, making protein carbonylation a reliable indicator of oxidative stress [10].

Specific proteins, such as chaperones, metabolic enzymes, photosynthetic proteins, and antioxidant enzymes, are at risk of carbonylation under these stress conditions, which can impact their functions and overall plant viability. The degradation of carbonylated proteins by the 20S proteasome generates peptides and free amino acids that can be reused for synthesising new proteins and stress-response enzymes, thereby supporting proteome homeostasis [8].

Two-dimensional electrophoresis (2D-PAGE) is a standard method for separating complex proteomes, facilitating the identification of stress-responsive proteins affected by post-translational modifications [11]. When combined with spectrophotometric and proteomic analyses of protein carbonylation, 2D-PAGE provides a comprehensive assessment of changes in the proteome under stress, revealing significant rearrangements and identifying proteins susceptible to oxidative modification [8].

Despite extensive research on potato responses to abiotic stresses, studies linking agronomic trait analysis with proteomic approaches and assessing oxidative protein modifications under varied stress conditions remain scarce. Integrating these levels of analysis is essential for linking molecular changes to overall plant performance. This study investigates stress-specific patterns of protein carbonylation and proteome remodelling in potato leaves subjected to drought, elevated temperature, and their combination. By combining biochemical and proteomic analyses with agronomic traits, this approach provides deeper insights into the molecular mechanisms underlying potato responses to complex abiotic stresses and identifies potential protein-level indicators of stress tolerance.

## 2. Results

### 2.1. Impact of Drought and Elevated Temperature on Leaf Water Status, Growth, and Yield

**Figure 1:**
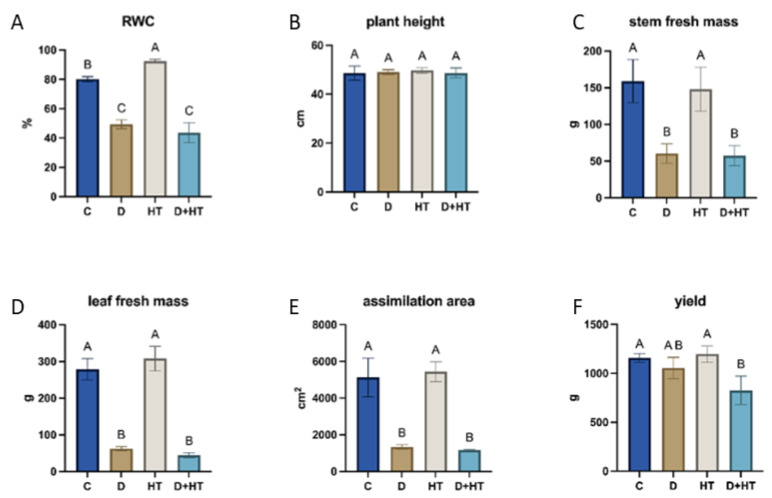
Plant parameters: relative water content (RWC; (**A**)), plant height (**B**), stem fresh mass (**C**), leaf fresh mass (**D**), assimilation area (**E**), and yield (**F**). Plants were maintained under control conditions (**C**) or subjected to drought (**D**), elevated temperature (HT), or their combined stress treatment (D+HT). Values are presented as means ± standard deviation (SD). Different letters denote statistically significant differences among treatments at *p* < 0.05, as determined by two-way ANOVA followed by Tukey’s post hoc test.

The RWC of potato leaves was strongly affected by the applied stresses (Figure 1A). Elevated temperature slightly increased RWC compared to the control, reaching about 92%. In contrast, drought caused a substantial decrease in RWC to approximately 49%, while drought and combined stress significantly reduced RWC to approximately 43%.

Plant height (Figure 1B) remained largely unaffected by stress treatments. No significant differences were observed among control, drought, elevated temperature, or combined stress conditions.

Stem fresh mass was significantly reduced under drought and combined stress conditions (Figure 1C). Drought and double stress decreased stem fresh mass by more than 60%, whereas elevated temperature reduced it, but not significantly.

Leaf fresh mass showed a similar pattern (Figure 1D). Elevated temperature did not significantly affect leaf biomass, while drought and combined stress caused a strong decrease in leaf fresh mass, reducing it by approximately 80% compared to the control.

The leaf assimilation area was also strongly affected by stresses (Figure 1E). Elevated temperature slightly increased the assimilation area, while drought and combined stress caused dramatic reductions of over 75%.

Overall, tuber yield was moderately affected by the applied stresses (Figure 1F). Elevated temperature and drought did not reduce yield, while combined stress led to a yield decrease of approximately 28% compared to the control.

### 2.2. Protein Carbonylation and Proteasome Activity

**Figure 2:**
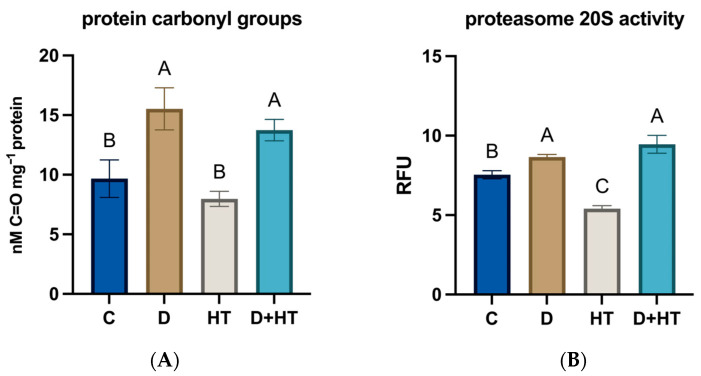
Protein carbonylation (**A**) and 20S proteasome activity (**B**). Plants were maintained under control conditions (C) or subjected to drought (D), elevated temperature (HT), or combined drought and high-temperature stress (D+HT). Values are presented as means ± standard deviation (SD). Different letters denote statistically significant differences among treatments at *p* < 0.05, as determined by two-way ANOVA followed by Tukey’s post hoc test.

Stress significantly affected protein carbonylation levels (Figure 2A). Exposure to elevated temperature resulted in an approximately 17% reduction in oxidised proteins compared to the control. In contrast, drought caused a pronounced increase of about 61%, and under combined stress, protein carbonylation also increased, reaching approximately 43% above control levels.

The 20S proteasome plays a vital role in removing carbonylated proteins via a ubiquitin-independent pathway. Proteasome activity decreased by approximately 30% under high-temperature stress, but increased by about 15% under drought, and by roughly 25% under combined stress compared to the control (Figure 2B).

### 2.3. Correlation Analysis of Morpho-Physiological and Biochemical Parameters

**Figure 3:**
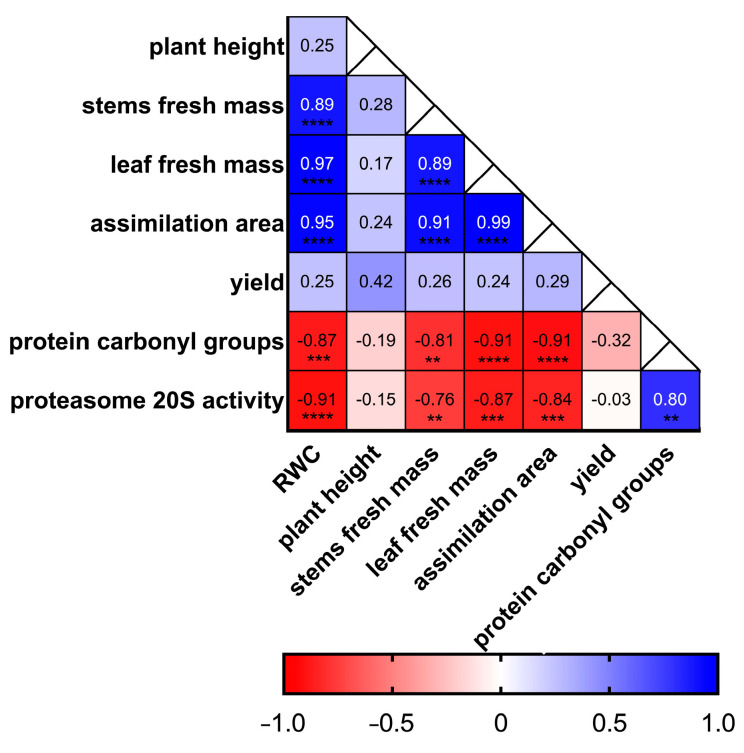
Heatmap of Pearson’s correlation coefficients between RWC, Plant Height, Stems and Leaf Fresh Mass, Assimilation Area, Yield, Protein Carbonyl Groups, and 20S Proteasome Activity. Dark blue indicates strong positive correlation, dark red strong negative correlation, and white near-zero correlation. Significance levels: **** *p*  ≤  0.0001, *** *p*  ≤  0.001, ** *p*  ≤  0.01.

Numerous interactions among the analysed parameters were observed (Figure 3). Very strong positive correlations (*p* ≤ 0.0001) were detected between RWC and Assimilation Area (0.95) and between RWC and Leaf Fresh Mass (0.97). Strong positive correlations were also found between Stem Fresh Mass and Leaf Fresh Mass (0.89, *p* ≤ 0.0001), Stem Fresh Mass and Assimilation Area (0.91, *p* ≤ 0.0001), and Protein Carbonyl Groups and 20S Proteasome Activity (0.80, *p* ≤ 0.01). A moderate positive correlation was observed between Yield and Plant Height (0.42).

The strongest negative correlations (*p* ≤ 0.0001) were recorded for RWC and 20S Proteasome Activity (−0.91), Leaf Fresh Mass and Protein Carbonyl Groups (−0.91), and Assimilation Area and Protein Carbonyl Groups (−0.91). Additional significant negative correlations were found between RWC and Protein Carbonyl Groups (−0.87, *p* ≤ 0.001) and Leaf Fresh Mass and 20S Proteasome Activity (−0.87, *p* ≤ 0.001). Lower negative correlations were observed between Yield and Protein Carbonyl Groups (−0.32).

### 2.4. Identification of Differentially Abundant and Carbonylated Proteins in Potato Leaves

Gels analysis revealed that out of 751 spots, the abundance of 21 was significantly altered in response to singular or combined stress.

Protein abundance ratios under combined stress, high temperature, and drought, relative to the control, are presented in Table 1. Several functional groups of proteins were distinguished based on their responses to stress.

Stress Response Proteins

Proteins associated with stress response exhibited the most pronounced changes, particularly under combined drought and temperature stress. The 20 kDa chaperonin showed the highest upregulation, with abundance ratios reaching 7.656 and 4.113 in the combined stress group. Chaperone protein ClpB3 also accumulated strongly under combined stress (5.224). Stromal 70 kDa heat shock-related protein and 2-methylene-furan-3-one reductase displayed strong responses under combined stress conditions, with the latter showing consistent upregulation across all treatments.

Carbohydrate, Energy, and Photosynthesis

Proteins involved in energy metabolism and photosynthesis exhibited distinct patterns. The ATP synthase gamma chain was significantly upregulated during drought (5.297) and combined stress (3.277), indicating a high demand for energy production. Partial isoforms of carbonic anhydrase showed substantial increases under drought conditions, with values as high as 5.955 and 4.823. In contrast, aconitate hydratase and several photosynthesis-related proteins, including RuBisCO activase and oxygen-evolving enhancer protein 1, were generally downregulated, particularly under combined stress conditions.

Protein and Amino Acid Metabolism

Proteins involved in synthesis and degradation displayed contrasting behaviours. Proteasome subunit beta type-6-like was strongly upregulated across all treatments, peaking at 4.742 in the drought group, suggesting an active role in degrading damaged proteins. Puromycin-sensitive aminopeptidase showed a specific and significant increase (3.276) under combined stress. However, elongation factor G was markedly downregulated, especially under combined and temperature stress (values near 0.05), indicating a substantial reduction in protein translation rates.

Others

Among other proteins, the uncharacterized protein LOC102578855/oxidoreductase showed a notable increase in response to temperature stress (3.123), while remaining low under drought or combined stress conditions.

Carbonylated protein abundance ratios under combined stress, high temperature, and drought, relative to the control, are presented in Table 2. Out of 701 protein spots, the abundance of 18 was significantly changed in response to stress. Different functional groups of proteins were identified.

Stress Response

The most prominent changes were observed among stress-responsive proteins, particularly molecular chaperones and antioxidant enzymes. Isoforms of the 20 kDa chaperonin exhibited extreme carbonylation upregulation, with one isoform increasing nearly 15-fold (14.959) in response to temperature stress, while another reached a ratio of 7.583 under combined stress conditions. L-ascorbate peroxidase (isoforms X1 and X2) and annexin were strongly carbonylated specifically under drought, with abundance ratios ranging from 3.325 to 3.984. Substantial accumulation of carbonylated heme-binding protein 2-like was also observed under both drought (3.833) and combined stress (1.952).

Photosynthesis and Energy Metabolism

Proteins associated with carbon fixation, glycolysis, and energy metabolism displayed fluctuations in carbonylation. The RuBisCO large subunit-binding protein alpha showed a pronounced 8.093-fold increase in carbonylation under combined stress, despite being downregulated under drought (0.478). Triosephosphate isomerase was significantly carbonylated during both drought (5.106) and combined stress (2.021). In contrast, glyceraldehyde-3-phosphate dehydrogenase and phosphoglycerate kinase showed lower carbonylation levels across all stress treatments, with the lowest values observed under combined stress. Glycine dehydrogenase carbonylation was also downregulated under both combined stress (0.281) and high-temperature (0.635) treatments.

Metabolism of Proteins, Amino Acids, and Nucleic Acids

Proteins involved in protein turnover and genetic regulation also exhibited significant changes. The carbonylation of proteasome-like protein alpha subunit was strongly induced under drought conditions (5.614), indicating an increased level of oxidative damage in proteins targeted for degradation. Within the nucleic acid metabolism category, the Agamous-like MADS-box protein AGL29 displayed a remarkable 7.958-fold increase in carbonylation specifically in response to drought, while carbonylated harpin-binding protein 1 also accumulated under the same condition (2.478).

### 2.5. Protein–Protein Interaction (PPI) Network

To investigate the potential functional interrelationships among the identified proteins, a protein–protein interaction (PPI) network was constructed utilizing the STRING database for Solanum tuberosum. The analysis identified several clusters of functionally related proteins, suggesting potential roles in analogous biological processes. It is important to note that the STRING database amalgamates evidence from a multitude of sources, including experimental data, computational prediction methods, co-expression analyses, text mining, and inferred interactions from other organisms via orthology.

Given the relatively limited availability of experimentally validated interaction data specific to *Solanum tuberosum*, a significant proportion of the interactions in the constructed network are likely extrapolated from interolog-based predictions derived from well-characterized model organisms. Therefore, the connections shown in the network are functional associations rather than direct physical protein–protein interactions. Furthermore, STRING networks serve as static, integrated interaction models that do not capture tissue specificity, developmental stages, or environmental variables that may significantly influence protein interactions in a physiological context.

**Figure 4:**
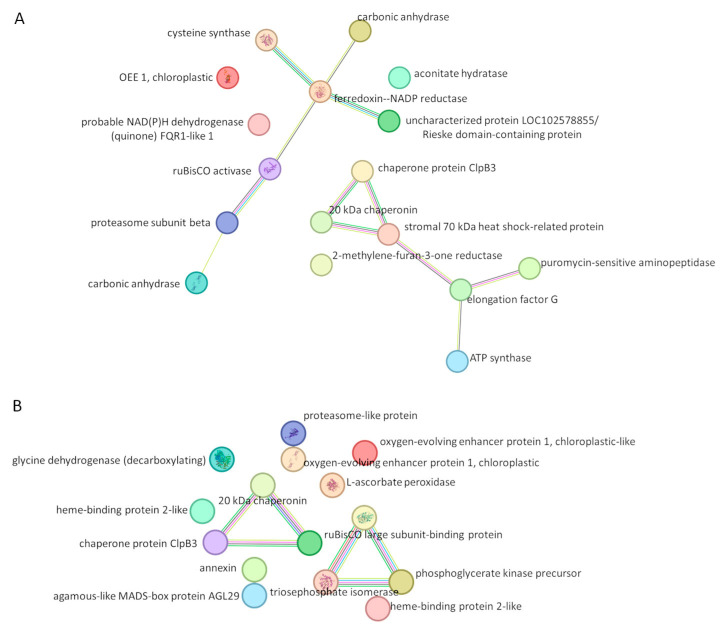
Protein interaction networks of differentially abundant proteins (**A**) and differentially carbonylated proteins (**B**) were generated using STRING version 12.0 (https://string-db.org accessed on 5 January 2026), a database of known and predicted protein–protein interactions (PPIs). Multiple proteins were analysed with the minimum interaction score set to 0.400. Edge colours indicate the type of interaction: known interactions—cyan (curated databases) and magenta (experimentally determined); predicted interactions—green (gene neighbourhood), red (gene fusions), and blue (gene co-occurrence); other evidence—lime (text mining), black (co-expression), and purple (protein homology).

STRING analysis of the differentially abundant proteins revealed an extensive network of interdependencies, suggesting a coordinated systemic response to the applied stress conditions (Figure 4A). A central hub was formed by ferredoxin–NADP reductase, which interacts with carbonic anhydrase, cysteine synthase, and RuBisCO activase, linking electron transport, primary metabolism, and proteasome function, while the uncharacterized Rieske domain-containing protein LOC102578855 indicates involvement in the photosynthetic electron transport chain. A distinct cluster of molecular chaperones and heat shock proteins, including ClpB3, the 20 kDa chaperonin, and stromal 70 kDa heat shock-related protein, highlights a coordinated stress-response system connected to the metabolic network via 2-methylene-furan-3-one reductase. Proteins involved in translation and energy production, such as elongation factor G, puromycin-sensitive aminopeptidase, and ATP synthase, form another interconnected group that bridges protein synthesis with cellular energy metabolism and maintains links to the chaperone cluster. Several proteins, including OEE 1 (chloroplastic), aconitate hydratase, and probable NAD(P)H dehydrogenase FQR1-like 1, appeared isolated, suggesting independent functions or roles in pathways not captured by the current interaction network.

Several interactions among the carbonylated proteins were observed (Figure 4B), revealing a structured network organized around protein folding and metabolic processes. A prominent cluster of molecular chaperones and folding-related proteins was identified, including the 20 kDa chaperonin, chaperone protein ClpB3, and the RuBisCO large subunit-binding protein, which together represent the plant’s machinery for maintaining protein stability and ensuring correct folding.

A second distinct hub was observed, centered on metabolic enzymes involved in energy production and carbon fixation, such as the RuBisCO large subunit-binding protein, phosphoglycerate kinase precursor, and triosephosphate isomerase. The RuBisCO large subunit-binding protein serves as a central bridge between the chaperone cluster and the metabolic enzymes, suggesting tight coordination between protein homeostasis and active photosynthesis/glycolysis.

Several other carbonylated proteins were detected but did not display direct interactions within this network. These include protective and antioxidant proteins (L-ascorbate peroxidase and chloroplastic oxygen-evolving enhancer protein 1), regulatory and transport proteins (annexin, heme-binding protein 2-like, and Agamous-like MADS-box protein AGL29), as well as metabolic enzymes (glycine dehydrogenase [decarboxylating] and a proteasome-like protein).

### 2.6. Integrative Heat Map Analysis of Differentially Abundant and Carbonylated Proteins

**Figure 5:**
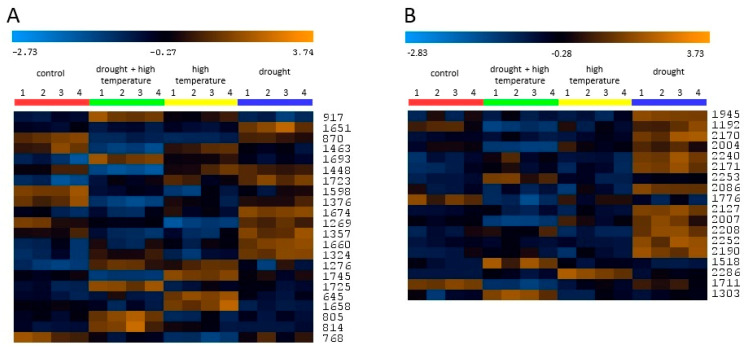
Comparative proteomic analysis of stress-responsive proteins. (**A**) Heatmap showing the profile of differentially abundant proteins under control and stress conditions. (**B**) Heatmap representing carbonylated proteins, highlighting those susceptible to oxidative damage. The colour scale indicates the relative abundance or carbonylation level (z-score), ranging from blue (decrease) to orange (increase). Numbers on the right represent unique protein identifiers (IDs).

Heatmap analysis of differentially abundant proteins revealed distinct changes corresponding to stress-specific responses (Figure 5A). Proteins strongly induced by drought, including carbonic anhydrase (IDs 1651, 1660, 1674), ATP synthase gamma chain (1324), and proteasome subunit beta type-6-like (1723), exhibited the highest abundance under drought conditions, whereas their levels decreased under high temperature. A separate group of proteins, upregulated specifically under combined stress, included 20 kDa chaperonin (1725, 1693), chaperone protein ClpB3 (814), and puromycin-sensitive aminopeptidase (805), highlighting a synergistic response not observed under individual stresses. Another group, downregulated across stress conditions, comprised elongation factor G (970), RuBisCO activase (1269), and aconitate hydratase (768), with the strongest repression under high temperature and combined stress. Finally, a high-temperature-specific protein, uncharacterized oxidoreductase (1658), was induced only by elevated temperature, while drought and combined stress decreased its abundance. Overall, these results indicate that drought primarily activates carbohydrate metabolism and proteolytic processes, whereas combined stress triggers a robust chaperone-mediated response to maintain protein stability under synergistic conditions.

Heatmap analysis of carbonylated proteins revealed distinct groups corresponding to stress-specific responses (Figure 5B). Proteins strongly carbonylated by drought, including Agamous-like MADS-box protein AGL29 (2252), triosephosphate isomerase (2171), proteasome-like protein alpha subunit (2170), annexin (1945), and L-ascorbate peroxidase cytosolic isoform X1 (2127), exhibited the highest abundance under drought conditions. In contrast, their levels were lower under high temperature. A separate group of carbonylated proteins, upregulated specifically under combined stress, included RuBisCO large subunit-binding protein subunit alpha (1518), 20 kDa chaperonin chloroplastic-like (2253), and chaperone protein ClpB3 chloroplastic (1303), highlighting a synergistic response not observed under individual stresses. Another group, with downregulated carbonylation across stress conditions, comprised glyceraldehyde-3-phosphate dehydrogenase (1711) and phosphoglycerate kinase precursor (1776), with the strongest repression of carbonylation under high temperature and combined stress. Finally, a high-temperature-specific protein, 20 kDa chaperonin chloroplastic-like (2286), was highly carbonylated only under elevated temperature, while drought and combined stress decreased its abundance. Overall, these results indicate that drought primarily changes metabolic and proteolytic pathways by carbonylation, whereas combined stress triggers a robust chaperone-mediated protective response, and high temperature specifically induces carbonylation of heat-responsive chaperones.

## 3. Discussion

Analysis of the results at the agronomic, biochemical, and proteomic levels shows that the potato’s response to environmental stress is complex. Furthermore, when multiple stresses occur, they are not the sum of responses to individual stresses. In temperate European climates, drought is often accompanied by high temperatures. Finding varieties that tolerate combinations of these unfavourable conditions is crucial, and understanding the mechanisms of potato response to combined stresses is essential. Even though the selected Lech potato variety is tolerant to high temperatures, in combination with drought, the negative effect on the yield was intensified.

### 3.1. Agronomic and Physiological Responses to Stress

Drought and high temperatures have significant impacts on plant water relations, which in turn disrupt photosynthesis and alter metabolic homeostasis. These stressors can lead to a reduction in biomass accumulation and tuber yield, as evidenced by various studies in the field [12,13]. Drought, whether applied alone or in combination with elevated temperatures, significantly decreases stem and leaf biomass, indicating that water availability is the main constraint on vegetative growth in potatoes. It is noteworthy that high temperatures tend to have minimal direct effects on these traits, underscoring the more dominant role of water deficiency. This phenomenon aligns with observations in potatoes and other crops, where water deficits lead to restricted cell expansion and hinder leaf development, primarily due to the loss of turgor pressure within the cells.

In contrast, moderate heat stress can be managed to some extent through plant acclimation mechanisms, such as the induction of heat shock proteins and enhanced membrane stabilization. These adaptive responses help to mitigate the negative effects of heat, allowing the plant to thrive despite elevated temperatures [14,15,16].

The reduction in leaf assimilation area under drought likely limits the plant’s capacity for light capture and carbon fixation, which underpins decreases in growth. Previous studies in potato, wheat, and maize have linked water limitation to diminished stomatal conductance, decreased intercellular CO_2_, and imbalances in photosynthetic energy partitioning [12,17,18]. Additionally, drought accelerates leaf senescence and reduces chlorophyll content, further limiting photosynthetic efficiency [17,19].

High temperatures did not significantly affect tuber yield; however, when combined with drought, they caused a noticeable decrease in yield. This suggests that yield loss arise mainly from simultaneous stress rather than from the additive effects of each stress.

### 3.2. Proteomic Responses: Differentially Abundant Proteins

Recent advances in proteomic profiling have provided new insights into the complex effects of stress on cellular protein dynamics. Increasing evidence suggests that physiological stress responses extend far beyond oxidative protein modifications, encompassing a comprehensive qualitative and quantitative reprogramming of the proteome [8]. Studies have identified significant upregulation of specific chaperone proteins in response to combined stress factors such as drought and elevated temperature. Notably, the 20 kDa chaperonin and ClpB3 demonstrated significant increases in abundance, indicating their critical role in the cellular stress response. Moreover, another noteworthy finding is the consistent accumulation of the stromal 70 kDa heat shock-related protein and 2-methylene-furan-3-one reductase across various stress treatments [8,20,21]. This pattern suggests a robust and coordinated response mechanism that facilitates protein stability and function under adverse environmental conditions. The substantial induction of these chaperones highlights their fundamental importance in maintaining cellular proteostasis, particularly in crops such as potato, Arabidopsis, and rice [22,23]. As plants face the dual challenges of drought and elevated temperature, the upregulation of these protective proteins is likely to mitigate the risks associated with the misfolding and aggregation of other stress-sensitive proteins. By doing so, they preserve essential metabolic activities and ensure the continued functionality of photosynthesis, which is vital for plant survival and productivity.

Proteins involved in photosynthesis and energy metabolism displayed stress-specific regulation. ATP synthase gamma chain was significantly upregulated under drought and combined stress, suggesting elevated energy demand for stress adaptation and repair processes. Partial isoforms of carbonic anhydrase showed pronounced accumulation under drought, potentially to maintain CO_2_ fixation under limited water availability. Conversely, RuBisCO activase, aconitate hydratase, and oxygen-evolving enhancer protein 1 were downregulated, likely contributing to the observed decreases in leaf assimilation area and photosynthetic capacity. These patterns are consistent with reports in maize and rice, where oxidative stress and drought induced downregulation of Calvin cycle and glycolytic enzymes, while certain compensatory energy-related proteins were upregulated [18,24].

Protein synthesis and degradation pathways were also strongly affected. The proteasome subunit beta type-6-like was upregulated in all treatments, particularly under drought, indicating enhanced proteolytic activity aimed at removing damaged or misfolded proteins. Puromycin-sensitive aminopeptidase showed significant accumulation under combined stress, suggesting targeted protein processing. In contrast, elongation factor G was severely downregulated in all treatments, indicating suppression of translation, which may contribute to reductions in growth and biomass. The coordination between reduced protein synthesis and increased degradation likely represents a protective strategy to maintain proteome integrity during stress [25,26].

### 3.3. Protein Carbonylation and Proteasome Activity Under Stress

Protein carbonylation is widely recognised as a marker of oxidative damage, arising from direct ROS attack on amino acid side chains or as a consequence of secondary reactions, e.g., with lipid peroxidation products [27,28]. In our experiments, both drought and combined stress markedly increased total protein carbonylation, indicating enhanced oxidative processes under water deficit. Elevated ROS production under drought is a well-established phenomenon, resulting from the disruption of electron transport in chloroplasts and mitochondria caused by stomatal closure, limited CO_2_ availability, and energy imbalance [29]. The simultaneous rise in 20S proteasome activity during drought and combined stress suggests that protein quality control systems are being activated to eliminate oxidatively damaged proteins. This finding aligns with previous observations in Arabidopsis and wheat, where abiotic stress has been shown to enhance proteolytic pathways [30]. This proteasome response likely contributes to maintaining proteome integrity by preventing the accumulation of dysfunctional proteins that could further impair cellular processes [31].

In contrast, high temperature reduced both protein carbonylation and proteasome activity, suggesting that short-term elevated temperature may trigger protective mechanisms that limit ROS accumulation. Previous studies indicate that heat stress can rapidly activate antioxidant systems and heat shock proteins, particularly during the early phase of exposure [32]. Drought caused pronounced protein carbonylation with only a moderate increase in proteasome activity, whereas combined drought and elevated temperature led to a lower increase in carbonylation accompanied by stronger proteasome activation. These results suggest that the simultaneous occurrence of both stresses does not produce a simple additive effect but rather triggers a distinct and complex regulation of oxidative damage and protein turnover, reflecting non-additive interactions between drought- and elevated temperature-responsive signaling pathways [8].

### 3.4. Carbonylated Proteome: Functional Implications

While global proteomic profiling captures quantitative remodeling of the proteome, analysis of the carbonylated proteome reveals the functional consequences of stress-induced oxidative damage, showing that proteins from multiple cellular pathways are selectively targeted by carbonylation [8].

Several stress-responsive proteins exhibited simultaneous overaccumulation and high carbonylation, highlighting the complex interplay between protective induction and oxidative damage. Molecular chaperones, including the 20 kDa chaperonin and ClpB3, were strongly upregulated in response to drought and combined stress, reflecting the urgent cellular demand for protein quality control. However, their simultaneous carbonylation suggests that these chaperones are themselves vulnerable to oxidative stress, potentially acting as sacrificial components to protect other metabolic and photosynthetic proteins [8,33].

Antioxidant enzymes, such as L-ascorbate peroxidase, were specifically targeted by carbonylation, particularly under drought conditions, despite showing limited changes in their global abundance. This suggests that impairment of the antioxidant system may result primarily from oxidative damage to existing enzymes rather than their reduced synthesis. At the same time, stress-responsive induction of antioxidant enzymes reflects an attempt to enhance ROS detoxification, although their carbonylation may compromise enzymatic activity and ultimately exacerbate oxidative stress [33,34]. Similarly, regulatory proteins like annexin, involved in membrane repair and ROS signaling, showed stress-dependent induction coupled with oxidative modification, indicating disruption of signalling pathways critical for stress adaptation [35,36,37].

Proteins involved in photosynthesis and energy metabolism exhibited a dual response under combined drought and elevated temperature. Notably, the RuBisCO large subunit-binding protein alpha was upregulated, suggesting an attempt by the plant to reinforce its photosynthetic machinery under adverse conditions. However, this protein also experienced carbonylation, which may hinder its chaperone function that is crucial for the proper assembly of RuBisCO, the primary enzyme responsible for carbon fixation [8,33].

Key metabolic enzymes, including glyceraldehyde-3-phosphate dehydrogenase and phosphoglycerate kinase, were simultaneously downregulated and oxidatively modified. This pattern mirrors observations in other crops, such as maize and rice, under combined drought and elevated temperature [38] indicating a broader impact on metabolic pathways critical for maintaining energy production under stress.

Additionally, the suppression of glycine dehydrogenase under elevated temperature highlights the disruption of the photorespiratory pathway. This enzyme catalyzes the conversion of glycolate to glycine in chloroplasts, a process that normally mitigates the effects of high O_2_ and low CO_2_ levels. Its inhibition can disturb energy balance and ROS homeostasis, leading to the accumulation of ROS, enhanced oxidative stress, and potential impairment of plant growth and productivity [8,39].

This integration of quantitative upregulation and oxidative modification underscores that increased protein abundance does not necessarily ensure functionality. The overproduction of chaperones and antioxidants may act as a compensatory mechanism to sustain minimal activity despite oxidative damage. Yet, prolonged or intensified stress can overwhelm this system, leading to the accumulation of dysfunctional, carbonylated proteins and hindered proteolytic pathways, including the 20S proteasome [40].

Altogether, these findings indicate that stress tolerance depends not only on the induction of protective proteins but also on maintaining their functional integrity, revealing the limits of cellular defense strategies under combined drought and elevated temperature.

## 4. Materials and Methods

### 4.1. Plant Material

The research was conducted at the Department of Potato Agronomy of the Plant Breeding and Acclimatization Institute—National Research Institute. The Polish potato cultivar *Solanum tuberosum* L. ‘Lech’, known for its sensitivity to soil drought, was used in the study. Plants were grown in 14 L pots under controlled conditions in a growth chamber. Each pot contained 12 L of a universal peat-based substrate (Hollas, Agaris Poland, Pasłęk, Poland) supplemented with chalk (pH 5.5–6.5). The substrate was enriched with NPK 14–16–18 fertilizer, providing 2.45 g of nitrogen, 1.22 g of phosphorus, and 2.61 g of potassium per plant.

High-quality seed tubers (3–4.5 cm in diameter) were pre-sprouted and planted at a depth of 5–6 cm. To improve soil aeration, each pot was equipped with a rubber tube. At the BBCH 20 growth stage, 10 g of MIS-3 fertilizer was applied per plant. Plants were watered daily to maintain the soil moisture content above 70% of the field water capacity. The growth chamber was maintained at a relative humidity of 65–70% and illuminated with six Hortilux Schreder lamps (1600 W each).

### 4.2. Stress Treatments

Two weeks after tuber initiation (approximately BBCH 40), plants were subjected to four experimental treatments for a duration of 14 days:

Control (C): Maintained optimal hydration (70% field water capacity, FWC) and standard temperature conditions (20/16 °C day/night).

Drought (D): Water withheld to reach and maintain 40% FWC at 20/16 °C.

Elevated Temperature (HT): Plants exposed to 38/25 °C (day/night) under optimal hydration (70% FWC).

Combined Stress (D+HT): Plants exposed to 38/25 °C (day/night) with water withheld to 40% FWC.

Representative photographic images illustrating the morphological appearance of plants under each treatment are provided in Supplementary Figure S1.

### 4.3. Sampling and Tissue Preparation

At the end of the 2-week stress period, fully expanded leaves from the third and fourth nodes (counting from the apex) were harvested for biochemical and proteomic analyses. For each biological replicate, six leaves were pooled; three independent biological replicates were performed per treatment. Collected leaf tissues were immediately flash-frozen in liquid nitrogen and ground into a fine powder using a chilled mortar and pestle to prevent protein degradation. Samples were stored at −80 °C until further analysis.

### 4.4. Agronomic Measurements

Yield: Tuber weight per plant measured at the end of vegetation.

Relative Water Content (RWC): Measured on 10 mature leaves per replicate using the formula [(FW − DW)/ (SW − DW)] × 100%.

Experimental Design: Conducted in two independent series, with six biological replicates per treatment.

Growth Traits: Plant height (cm), leaf area (cm^2^) and the dry mass (g) of stems, leaves were recorded post-stress.

Dry Mass Determination: Individual organs were dried in a two-stage process (24 h at 75 °C, then 105 °C until constant weight).

Leaf Area: Measured for all compound leaves per plant using an LI-3100A area meter (LI-COR, Lincoln, NE, USA).

### 4.5. Biochemical Assays

Protein carbonylation was determined according to Levine et al. (1994) [41] using DNPH (2,4-dinitrophenylhydrazine) derivatization. The absorbance was measured at 375 nm, and results were expressed as nmol carbonyl per mg of protein. The 20S proteasome activity was measured fluorometrically using 5 µg of total protein with a commercial assay kit (MAK172, Sigma-Aldrich, Saint Louis, MI, USA), following the manufacturer’s instructions.

Total Protein Extraction. Leaf tissue (150 mg) was ground in liquid nitrogen, and proteins were precipitated with ice-cold 10% TCA/acetone. The resulting pellet was washed sequentially with 10% TCA/acetone, 80% methanol containing 0.1 M ammonium acetate, and 80% acetone. The dried pellet was resuspended in a mixture of Tris-buffered phenol (pH 8.0) and SDS extraction buffer (0.1 M Tris-HCl, 30% sucrose, 5% β-mercaptoethanol, 2% SDS). After centrifugation, the phenolic phase was collected. Proteins were precipitated overnight with 0.1 M ammonium acetate in 80% methanol at −20 °C, washed with methanol and acetone, and finally dissolved in IEF buffer (7 M urea, 2 M thiourea, 4% CHAPS, 40 mM DTT). Protein concentration was determined using the Bradford-based method adapted for proteomic samples [42].

Two-Dimensional Gel Electrophoresis (2D-PAGE) and Immunoblotting. For proteome mapping, 120 µg of protein was loaded onto 7 cm pH 4–7 NL Immobiline DryStrips. Following isoelectric focusing (IEF), strips were equilibrated in buffers containing DTT (reduction) and iodoacetamide (alkylation) and separated on 11% SDS-PAGE gels. Gels were stained with colloidal Coomassie Brilliant Blue G-250.

For carbonylation detection, 20 µg of protein was derivatized with DNPH before IEF. After 2D separation, proteins were electro-transferred to PVDF membranes. Membranes were blocked with 5% non-fat milk and incubated with primary anti-DNP antibodies (Sigma-Aldrich), followed by alkaline phosphatase-conjugated secondary antibodies. Signals were visualized using NBT/BCIP substrate, and the reaction was terminated with distilled water.

### 4.6. Proteome Profiles Analysis

Four biological replicates were conducted for each experimental variant, including protein purification, two-dimensional electrophoresis (2DE), membrane transfer, and immunochemical staining (*n*  =  4). Gels and PVDF membranes were scanned using an Image Scanner III (GE Healthcare, Chicago, IL, USA), and their digital images were analyzed with Delta2D Version 2.0 software (DECODON GmbH, Greifswald, Germany).

Differential proteins were identified by comparing plants subjected to drought, high temperature, and a combination of these stresses with control leaves from the same culti-var. After correcting for positional spot variations, a virtual fused image was created to consolidate the information from all images into one composite image, followed by the detection of a consensus spot pattern. For spot quantification based on size and intensity, a standard procedure embedded in the software was applied to all membrane images from the experiment.

Normalized spot intensities were calculated by relating the intensity of each individual spot to the total intensity of all detected spots in the gel or membrane image. The accuracy of gel or membrane identity between biological replicates was assessed using principal component analysis (PCA), which served as both a quality control step and a means of comparing all experimental variants. The selection of differential proteins was based on mean spot intensity and was evaluated using one-way ANOVA with an adjust-ed Bonferroni correction (critical *p*-value  <  0.01). Images of selected spots on membranes were overlaid onto the corresponding gel images, and the selected spots were then excised from the gels for identification purposes.

### 4.7. Protein Identification and Bioinformatic Analysis

Differential spots were excised, destained, and subjected to in-gel trypsin digestion. Peptides were analyzed by LC–MS/MS using an Orbitrap Exploris 480 (Thermo Fisher Scientific, Waltham, MA, USA). MS/MS spectra were searched against the *Solanum tuberosum* NCBI database using the MASCOT engine (Matrix Science, London, UK). Search parameters included: ±5 ppm peptide tolerance, ±0.01 Da fragment tolerance, and two missed cleavages. Carbamidomethylation was set as a fixed modification, while methionine oxidation was considered a variable modification. Functional protein interaction networks were predicted using STRING 12.0 (minimum score 0.4).

### 4.8. Statistical Analysis

Data are presented as means ± SD. A two-way ANOVA followed by Tukey’s post hoc test (*p* < 0.05) was used to determine significant differences. Variance homogeneity was verified using the Brown-Forsythe test. Pearson’s correlation coefficients were calculated to evaluate relationships between traits. All analyses were performed using GraphPad Prism 10.

## 5. Conclusions

The differential responses to drought and elevated temperature reveal distinct yet overlapping signaling networks that influence plant stress adaptation. Drought primarily triggers abscisic acid (ABA) responses, affecting stomatal closure, antioxidant defenses, and alternation of expression of genes related to dehydration tolerance [43]. In contrast, high temperatures activate heat shock factor (HSF) pathways, inducing heat shock proteins that stabilize denatured proteins [44].

Our findings indicate drought as the main cause of oxidative protein damage, while high temperatures exacerbate this damage in combination with drought. The overaccumulation and carbonylation of stress-responsive proteins underline the importance of preserving functional integrity for stress tolerance. While protective proteins increase, their oxidative modifications reveal limitations of cellular defences under prolonged stress. These insights enhance our understanding of drought and elevated temperature responses in potato, providing potential targets for breeding and biotechnological intervention. Future research integrating transcriptomics and metabolic profiling will be crucial for identifying biomarkers of stress tolerance and developing resilient cultivars.

## Figures and Tables

**Table 1:** Identification of differentially abundant proteins in *Solanum tuberosum* L. leaves using LC–MS/MS analysis. Abbreviations: No.—spot number; Ratio—relative abundance of proteins under drought (D), elevated temperature (HT), and their combination (D+HT) compared to control (C) conditions; Seq.—number of identified sequences; emPAI—exponentially modified protein abundance index; pI—theoretical isoelectric point; Cov.—sequence coverage (%); FDR—False Discover Rate (%). Proteins were considered confidently identified when the Mascot score exceeded the significance threshold (*p* < 0.05) and the peptide FDR was below 5%. Changes in protein abundance were considered relevant when the fold change was ≥1.5 or ≤0.67.

No	Protein	Ratio D+HT/C	Ratio HT/C	Ratio D/C	Accession	Score	Matches	Seq	emPAI	Mass	pI	Cov	FDR
carbohydrate and energy metabolism													
768	aconitate hydratase, cytoplasmic	0.518	0.281	0.689	NP_001412306.1	1200	29 (29)	21 (21)	1.27	108,955	7.29	21	1.43
1324	PREDICTED: ATP synthase gamma chain, chloroplastic	3.277	1.186	5.297	XP_006348117.1	1048	19 (19)	11 (11)	2.05	41,623	7.56	29	0.93
1745	PREDICTED: probable NAD(P)H dehydrogenase (quinone) FQR1-like 1	0.305	2.143	1.124	XP_006362921.1	310	5 (5)	5 (5)	1.62	21,710	6.21	25	3.12
photosynthesis													
1269	PREDICTED: ribulose bisphosphate carboxylase/oxygenase activase, chloroplastic	0.610	0.223	1.018	XP_006349493.1	1690	37 (37)	21 (21)	5.86	48,374	8.10	51	0.65
1376	PREDICTED: ferredoxin-NADP reductase, leaf-type isozyme, chloroplastic	0.353	0.637	0.791	XP_006340740.1	2122	57 (57)	23 (23)	15.47	40,717	8.37	49	1.79
1448	Oxygen-evolving enhancer protein 1, chloroplastic	0.370	1.084	1.101	P26320.1	2142	50 (50)	18 (18)	7.43	35,595	5.84	57	1.11
1463	PREDICTED: oxygen-evolving enhancer protein 1, chloroplastic	0.269	0.929	0.568	XP_006344816.1	1965	46 (46)	16 (16)	6.69	35,168	5.91	48	1.54
1598	PREDICTED: carbonic anhydrase, chloroplastic	0.448	0.336	0.528	XP_006338560.1	257	7 (7)	5 (5)	0.85	36,369	6.71	15	4.17
1651	carbonic anhydrase, partial	0.405	0.74	5.955	BAM15483.1	970	20 (20)	12 (12)	3.26	34,919	6.71	40	1.64
1660	carbonic anhydrase, partial	1.660	1.692	4.246	BAM15483.1	878	18 (18)	12 (12)	3.24	34,919	6.40	41	2.04
1674	carbonic anhydrase, partial	0.830	1.775	4.823	BAM15483.1	802	16 (16)	10 (10)	2.34	34,919	6.40	33	1.73
protein and amino acid metabolism													
805	PREDICTED: puromycin-sensitive aminopeptidase isoform X1	3.276	0.526	0.777	XP_006343961.1	1370	35 (35)	28 (28)	2.19	111,526	6.07	26	0.98
970	PREDICTED: elongation factor G, chloroplastic	0.053	0.048	0.690	XP_006355498.1	1141	23 (23)	19 (19)	1.54	86,712	5.40	26	2.33
1357	cysteine synthase	0.341	0.468	1.602	NP_001274978.1	793	14 (14)	9 (9)	2.02	34,401	5.93	36	1.96
1723	PREDICTED: proteasome subunit beta type-6-like	2.463	3.314	4.742	XP_006365635.1	801	12 (12)	8 (8)	3.44	25,355	5.51	36	1.15
stress response													
814	PREDICTED: chaperone protein ClpB3, chloroplastic	5.224	1.722	0.850	XP_006338388.1	1094	32 (32)	24 (24)	1.52	110,594	6.17	24	1.85
917	PREDICTED: stromal 70 kDa heat shock-related protein, chloroplastic	2.153	1.438	0.784	XP_006349319.1	2596	48 (48)	28 (28)	3.86	75,217	5.16	37	1.92
1276	PREDICTED: 2-methylene-furan-3-one reductase	1.645	1.626	1.218	XP_006365236.1	2531	48 (48)	20 (20)	6.89	41,008	6.48	50	0.65
1693	PREDICTED: 20 kDa chaperonin, chloroplastic-like/Chaperonin 21	4.113	2.482	1.777	XP_006353790.1	1796	32 (32)	13 (13)	6.70	26,601	6.85	63	0.66
1725	REDICTED: 20 kDa chaperonin, chloroplastic-like/Chaperonin 21	7.656	1.615	1.835	XP_006345251.1	786	16 (16)	10 (10)	3.85	26,668	7.79	46	1.20
others													
1658	PREDICTED: uncharacterized protein LOC102578855/oxidoreductase	0.419	3.123	0.307	XP_006363858.1	859	16 (16)	9 (9)	2.51	30,224	6.12	36	1.85

**Table 2:** Identification of differentially carbonylated proteins in *Solanum tuberosum* L. leaves via LC–MS/MS. Abbreviations: No.—spot number; Ratio—comparative level of protein carbonylation in drought (D), elevated temperature (HT), and combined stress (D+HT) relative to control (C) samples; Seq.—number of peptide sequences; emPAI—exponentially modified protein abundance index; pI—isoelectric point; Cov.—sequence coverage (%); FDR—False Discover Rate (%). Proteins were considered confidently identified when the Mascot score exceeded the significance threshold (*p* < 0.05) and the peptide FDR was below 5%. Changes in protein abundance were considered relevant when the fold change was ≥1.5 or ≤0.67.

No	Protein	Ratio D+HT/C	Ratio HT/C	Ratio D/C	Accession	Score	Matches	Seq	emPAI	Mass	pI	Cov	FDR
carbohydrate and energy metabolism													
1711	glyceraldehyde-3-phosphate dehydrogenase (chloroplast)	0.111	0.375	0.628	CBL43264.1	2048	31 (31)	16 (16)	3.02	48,538	7.06	35	1.45
1776	phosphoglycerate kinase precursor	0.314	0.694	0.449	AAC26785.1	4312	79 (79)	30 (30)	11.20	50,594	7.68	73	0.60
2171	PREDICTED: triosephosphate isomerase, chloroplastic-like	2.021	1.180	5.106	XP_006367334.1	677	12 (12)	11 (11)	2.85	34,374	6.66	36	2.38
photosynthesis													
1518	PREDICTED: ruBisCO large subunit-binding protein subunit alpha	8.093	1.394	0.478	XP_006340213.1	2291	68 (56)	29 (27)	5.31	61,974	5.37	53	0.78
2004	REDICTED: oxygen-evolving enhancer protein 1, chloroplastic-like	0.183	1.045	1.497	XP_006338257.1	683	43 (28)	20 (16)	5.65	35,543	5.84	62	0.83
2007	PREDICTED: oxygen-evolving enhancer protein 1, chloroplastic	0.305	1.236	1.986	XP_006344816.1	1439	31 (31)	17 (17)	7.57	35,168	5.91	55	1.02
protein and amino acid metabolism													
1192	glycine dehydrogenase (decarboxylating), mitochondrial	0.281	0.635	1.172	NP_001305600.1	1029	47 (38)	24 (22)	1.27	113,868	6.52	22	0.93
2170	proteasome-like protein alpha subunit	0.857	1.007	5.614	NP_001275440.1	772	13 (13)	9 (9)	2.98	27,293	5.63	39	1.67
nucleic acid metabolism													
2086	harpin binding protein 1	0.724	0.792	2.478	NP_001274864.1	1268	20 (20)	10 (10)	3.00	30,301	8.31	43	2.44
2252	REDICTED: agamous-like MADS-box protein AGL29	1.181	0.745	7.958	XP_015158947.1	47	1 (1)	1 (1)	0.25	18,950	6.25	4	4.97
stress response													
1303	PREDICTED: chaperone protein ClpB3, chloroplastic	3.325	0.947	1.169	XP_006338388.1	2700	52 (52)	36 (36)	2.97	110,594	6.17	40	1.28
1945	annexin, partial	0.736	0.597	3.325	QBF76336.1	1105	31 (28)	18 (17)	8.42	31,833	6.57	61	0.91
2127	PREDICTED: L-ascorbate peroxidase, cytosolic isoform X1	0.888	0.910	3.544	XP_006361766.2	540	12 (12)	8 (8)	2.35	27,796	5.63	37	1.75
2190	PREDICTED: L-ascorbate peroxidase 2, cytosolic isoform X2	1.399	0.879	3.984	XP_015170961.1	952	18 (18)	11 (11)	4.68	27,263	5.87	48	1.79
2208	PREDICTED: heme-binding protein 2-like	1.952	0.489	3.833	XP_006348362.1	532	9 (9)	6 (6)	1.61	26,148	8.20	30	3.03
2240	PREDICTED: 20 kDa chaperonin, chloroplastic-like/chaperonin 21	3.223	1.142	6.836	XP_006345251.1	709	14 (14)	10 (10)	3.85	26,668	7.79	46	1.43
2253	PREDICTED: 20 kDa chaperonin, chloroplastic-like/chaperonin 21	7.583	1.279	2.483	XP_006353790.1	1538	26 (26)	13 (13)	7.84	26,601	6.85	56	1.56
2286	PREDICTED: 20 kDa chaperonin, chloroplastic-like/chaperonin 21	2.527	14.959	3.332	XP_006345251.1	914	20 (20)	10 (10)	3.85	26,668	7.79	44	1.05

## Data Availability

The original data presented in the study are openly available in the ProteomeXchange Consortium via PRIDE (https://doi.org/10.6019/PXD075691; Project accession: PXD075691).

## Supplementary Materials

The following supporting information can be downloaded at: https://www.mdpi.com/article/10.3390/plants15060939/s1, Figure S1: photographic images illustrating the morphological appearance of plants.
