# Supplementary figures and images for: Stress-Specific Carbonylation and Proteasome 20S Activity in Potato Under Drought, Elevated Temperature, and Combined Stresses: Linking Oxidative Damage to Proteome Regulation
Source: Plants (Basel). 2026 Mar 19;15(6):939. doi: 10.3390/plants15060939 (PMC13030461; doi:10.3390/plants15060939)

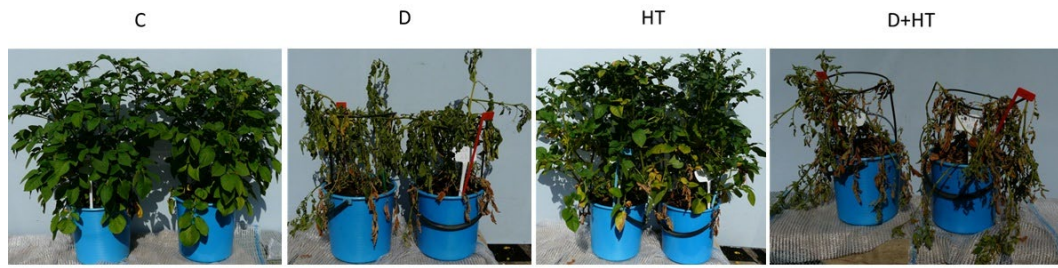

**Figure S1.** photographic images illustrating the morphological appearance of plants.

Supplement: Supplementary file 1 [file plants-15-00939-s001.zip › plants-4195220-supplementary.pdf]
